# Regulation of the IGF1 signaling pathway is involved in idiopathic pulmonary fibrosis induced by alveolar epithelial cell senescence and core fucosylation

**DOI:** 10.18632/aging.203335

**Published:** 2021-07-30

**Authors:** Wei Sun, Xiaoyan Jing, Xiaoyu Yang, Hui Huang, Qun Luo, Shu Xia, Ping Wang, Na Wang, Qian Zhang, Jian Guo, Zuojun Xu

**Affiliations:** 1 Department of Respiratory and Critical Medicine, Peking Union Medical College Hospital, Chinese Academy of Medical Sciences and Peking Union Medical College, Beijing, China; 2 Medical Research Center, Peking Union Medical College Hospital, Chinese Academy of Medical Sciences and Peking Union Medical College, Beijing, China; 3 State Key Laboratory of Respiratory Disease, National Clinical Center for Respiratory Disease, Guangzhou Institute of Respiratory Health, The First Affiliated Hospital of Guangzhou Medical University, Guangzhou, China

**Keywords:** IPF, aging, alveolar epithelial cell, core fucosylation, IGF-1

## Abstract

Idiopathic pulmonary fibrosis (IPF) mainly occurs in elderly people over the age of sixty. IPF pathogenesis is associated with alveolar epithelial cells (AECs) senescence. Activation of PI3K/AKT signaling induced by insulin-like growth factor 1 (IGF1) participates in AEC senescence and IPF by releasing CTGF, TGF-β1, and MMP9. Our previous study demonstrated that core fucosylation (CF) modification, catalyzed by a specific core fucosyltransferase (FUT8) can regulate the activation of multiple signaling pathways, and inhibiting CF can alleviate pulmonary fibrosis in mice induced by bleomycin. However, whether CF is involved in IGF1-mediated AEC senescence in IPF remains unclear. In this study, we found that the IGF1/PI3K/AKT signaling pathway was activated in IPF lung tissue. Meanwhile, CF was present in senescent AECs. We also showed that IGF1 could induce AECs senescence with enhanced CF *in vivo* and *in vitro*. Inhibiting CF alleviated AECs senescence and pulmonary fibrosis induced by IGF1. In addition, activation of IGF1/PI3K/AKT signaling depends on CF. In conclusion, this study confirmed that CF is an important target regulating the IGF1 signaling pathway in AEC senescence and IPF, which might be a candidate target to treat IPF in the future.

## INTRODUCTION

Idiopathic pulmonary fibrosis (IPF) is a refractory disease with unknown etiology, and the survival time after diagnosis is less than 3 years [1]. Notably, IPF occurs mainly in the elderly population [2]. Although morbidity and mortality increase with age, no effective treatment has been developed to prevent IPF progression [3].

Compromised self-repair function of alveolar epithelial cells (AECs) after exposure to a micro-injury environment is thought to initiate IPF [4]. Recently, senescence was identified as an important cause of the dampened AECs repair function in IPF, resulting decreased AECs regeneration and repair [5, 6]. Currently, the cause and regulatory mechanism of AECs senescence are unclear. Recent studies have found that insulin-like growth factor 1 (IGF1) is an important factor that increases cell senescence [7]. IGF1 is a natural growth hormone that plays an important role in the growth and development of the human body [8]. Under pathological conditions, AECs release IGF1, which activates the IGF receptor (IGFR-1) on adjacent normal AECs surfaces, and further activates intracellular downstream phosphoinositide 3-kinase (PI3K) and protein kinase B (PKB, also known as AKT) [9–11]. Eventually, cell senescence occurs by inducing the overexpression of P21 and P16 (cyclin dependent kinase inhibitor 1A and cyclin dependent kinase inhibitor 2A, respectively), both of which are key proteins of senescence [8, 12, 13]. Profibrotic cytokines such as connective tissue growth factor (CTGF), transforming growth factor-β (TGF-β), and matrix metalloproteinases (MMPs), are associated with AECs senescence [14–16]. These cytokines promote inflammatory cell infiltration, which sustain fibroblasts activation and differentiation to myofibroblasts, leading to collagen and extracellular matrix (ECM) synthesis [17]. Regulation of the IGF1 signaling pathway activity might restore the repair function of AECs and inhibit the IPF progress. Considering the important role of the IGF1/PI3K/AKT signaling pathway in inducing AECs senescence in pulmonary fibrosis, determining the mechanisms that regulate this signaling pathway is imperative.

After being synthesized, proteins need to undergo certain processes for their functionalization [18]. The core fucosylation (CF) modification is an important protein modification that is catalyzed by α1,6-fucosyltransferase (FUT8) in the Golgi apparatus, which adds fucose to the innermost GlcNAc residue of N-linked oligosaccharides on glycoproteins [19]. This modification is recognized preferentially by Lens culinaris lectin (LCA). Insulin-like growth factor receptor 1 (IGFR-1), which recognizes and initiates the downstream IGF1 signaling pathway, is a glycoprotein with post-translational glycosylation modification [20]. Moreover, our previous study demonstrated that many key proteins including transforming growth factor beta receptor (TGFβR) and platelet derived growth factor receptor beta (PDGFβR) were modified by CF [21]. CF also plays an important role in the Bleomycin (BLM)-induced mouse model of pulmonary fibrosis [22]. Therefore, we hypothesized that CF may be an important target for the precise regulation of the IGF1 signaling pathway, which could block the IPF process induced by AEC senescence.

In the present study, we observed activation of the IGF1/PI3K/AKT signaling pathway in the lung tissue of patients with IPF and upregulation of CF levels in AECs senescence. IGF1 can induce mouse AECs senescence together with an elevated level CF *in vitro*. Downregulation of CF prevented the AECs senescence induced by IGF1 by inhibiting the activities of the IGF1/PI3K/AKT signaling pathway. These data demonstrated that CF might be a potential target for IPF treatment by regulating the AECs senescence induced by IGF1 signaling pathway activation.

## RESULTS

### Activation of IGF1 signaling pathway in lung tissue of patients with IPF

**Figure 1:**
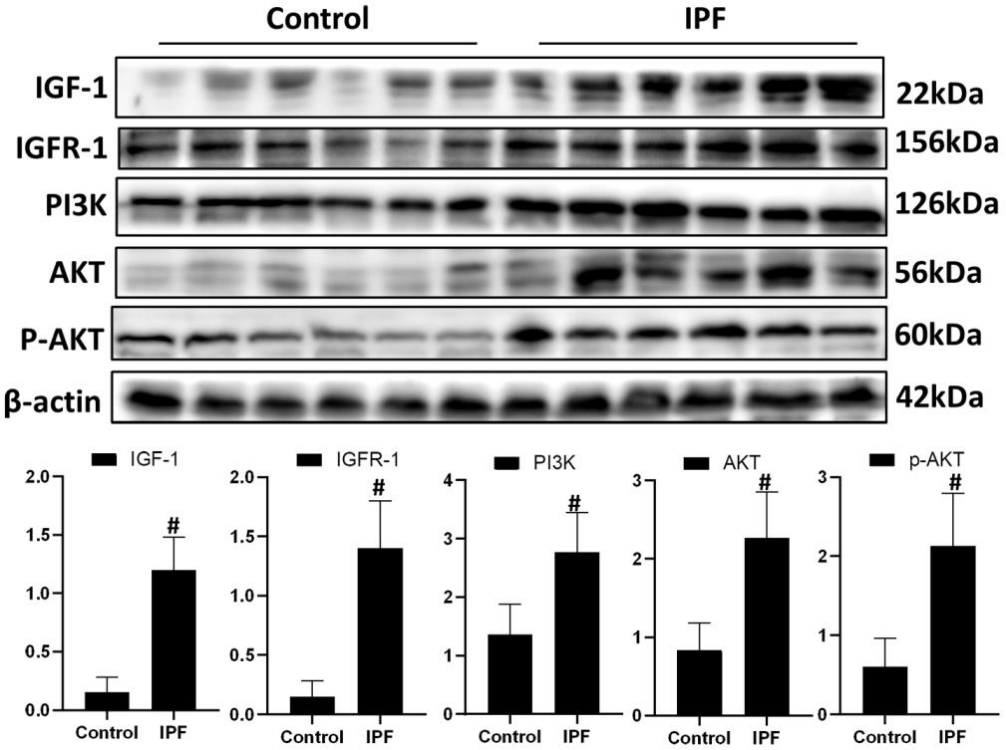
**Activated IGF1 pathway in lung tissue from patients with IPF.** IGF-1, IGFR-1, PI3K, AKT and p-AKT were assessed using western blotting analyses. #*P* < 0.01 for the comparison between the control group with the IPF group. Unpaired, two-tailed Student’s t test.

To evaluate the role of IGF1 in IPF, lung tissue from patients with IPF were analyzed for the levels of IGF1 and IGF1/PI3K/AKT signaling pathway members. Western blotting showed that the levels of IGF1, IGFR-1, PI3K, AKT and p-AKT in lung tissues of patients with IPF were significantly higher than those in normal lung tissues (Figure 1). This indicated that the IGF1 signaling pathway is activated in IPF conditions.

### AECs senescence in IPF lung tissue is accompanied by CF modification

**Figure 2:**
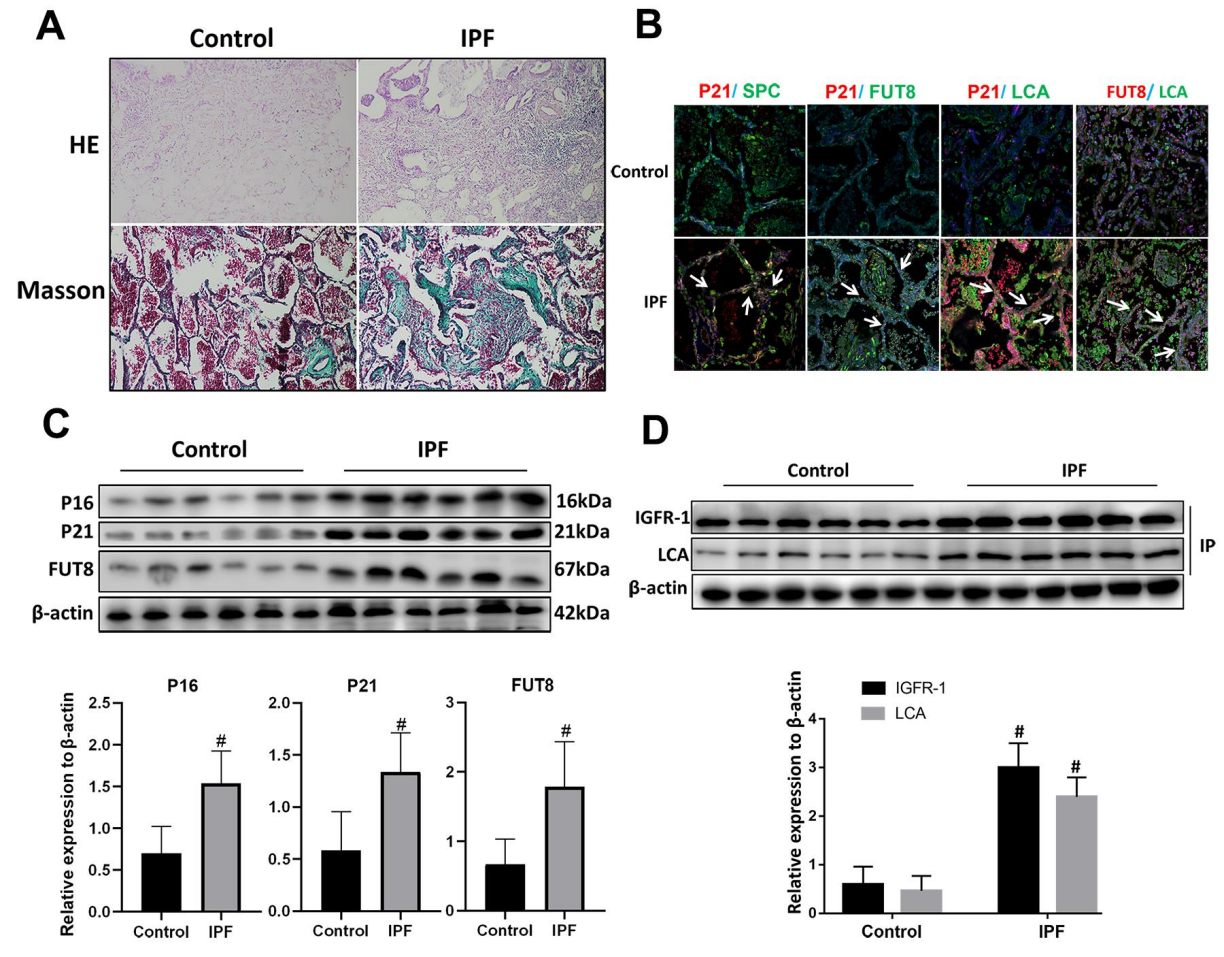
**CF was increased in AECs of lung tissue from patients with IPF.** (**A**) Representative results of HE staining and Masson staining in normal lung and lungs of patients with IPF (original magnification, 200×). (**B**) Representative images of dual staining for SPC (green) and P21 (red), FUT8 (green) and P21 (red), LCA (green) and P21 (red), and LCA (green) and FUT8 (red) (original magnification, 200×). (**C**) Western blotting was applied to detect the levels of activated P21, P16, and FUT8. (**D**) Lectin blot analysis of the immunoprecipitated IGFR-1 protein. IGFR-1 was immunoprecipitated from whole cell lysates using anti-IGFR-1 antibodies. The blots were probed with LCA. Representative data are shown. Quantification is shown in the lower panel. #*P* < 0.01 for the comparison between the control group and the IPF group. Unpaired, two-tailed Student’s t test.

Hematoxylin and eosin (HE) and Masson staining showed that alveolar structure was destroyed and blue stained collagen fibers appeared in the interstitium of the lung parenchyma from patients with IPF compared with that in normal lung tissues (Figure 2A). Immunofluorescence showed that there were significantly increased expression of senescence key protein (P21) in AECs and the increased p21 were accompanied by up-regulated CF (as indicated by FUT8 and LCA levels) (Figure 2B). Western blotting also showed that the levels of senescence key protein (P21, P16) and FUT8 were upregulated in IPF lung tissue (Figure 2C). Immunoprecipitation showed that LCA of IGFR-1 increased significantly in IPF lung tissue (Figure 2D). These results indicated that IPF is associated with AECs senescence, and AECs senescence is accompanied by CF.

### IGF1 induces enhanced CF in AECs senescence

**Figure 3:**
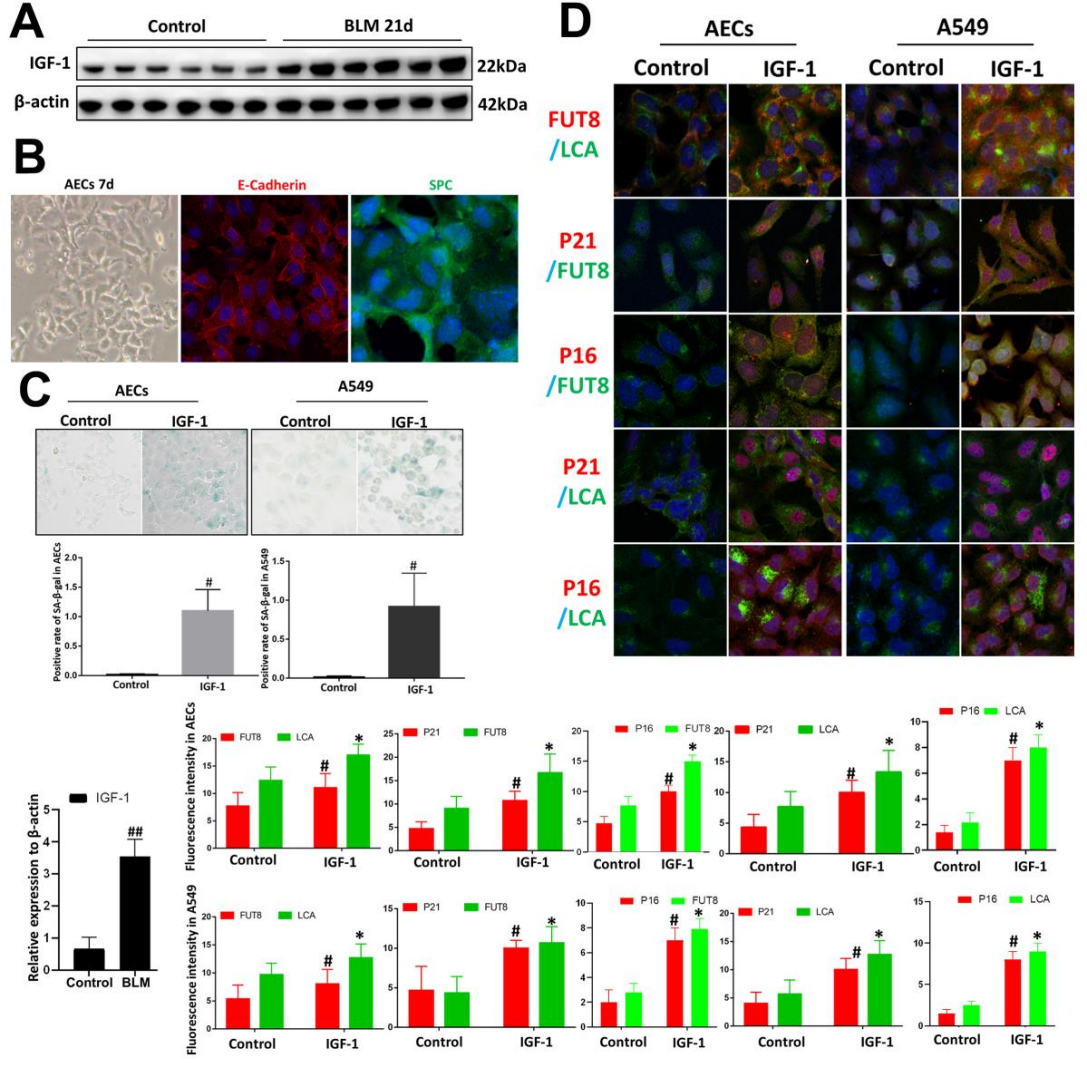
**Core fucosylation was increased during the IGF1-induced AEC senescence *in vitro*.** Primary cultures of AECs and A549 cells were incubated with IGF-1 (10 ng/ml) for 72 h. (**A**) The level of IGF1 was assessed using western blotting in different groups. (**B**) Representative bright-field images in AECs, and representative images of E-cadherin (red), SPC (green) staining is shown (original magnification, 200×). (**C**, **D**) SA-β-gal staining and representative images of dual staining for LCA (green) and FUT8 (red), FUT8 (green) and P21 (red), FUT8 (green) and P16 (red), LCA (green) and P21 (red) and LCA (green) and P16 (red) were performed to detect cellular senescence (original magnification, 200×). Data are shown as the mean ± SEM, n ≥ 3 per group. ##*P* < 0.01 for the comparison between the control group and the BLM group. *P < 0.01, #*P* < 0.01 for the comparison between the control group and the IGF1 group. Unpaired, two-tailed Student’s t test.

We used BLM mouse model to evaluate the expression of IGF1. Western blotting showed that the level of IGF1 in the lung tissue of BLM mice was significantly upregulated than that of the control group (Figure 3A). To reveal whether CF is involved in IGF1-induced AECs senescence, we extracted primary mouse AECs; the human type II alveolar epithelial cell line A549 was selected as the control. The type II AEC-specific marker surfactant protein C (SPC) and E-Cadherin were analyzed to verify the extracted primary AECs (Figure 3B). IGF1 (10 ng/ml) was used to induce the AEC senescence. Senescence-associated beta-galactosidase (SA-β-Gal) staining showed that after 72 h of IGF1 induction, the rate of positive cells increased significantly, indicating a cellular senescence phenotype induced by IGF1(Figure 3C). Immunofluorescence also showed that P21, P16 levels were upregulated after IGF1 induction. FUT8 and LCA levels were also upregulated, indicating an elevated level of CF (Figure 3D). These results demonstrated enhanced CF in AECs senescence induced by IGF1.

### Downregulation of CF rescues AECs from senescence induced by IGF1

**Figure 4:**
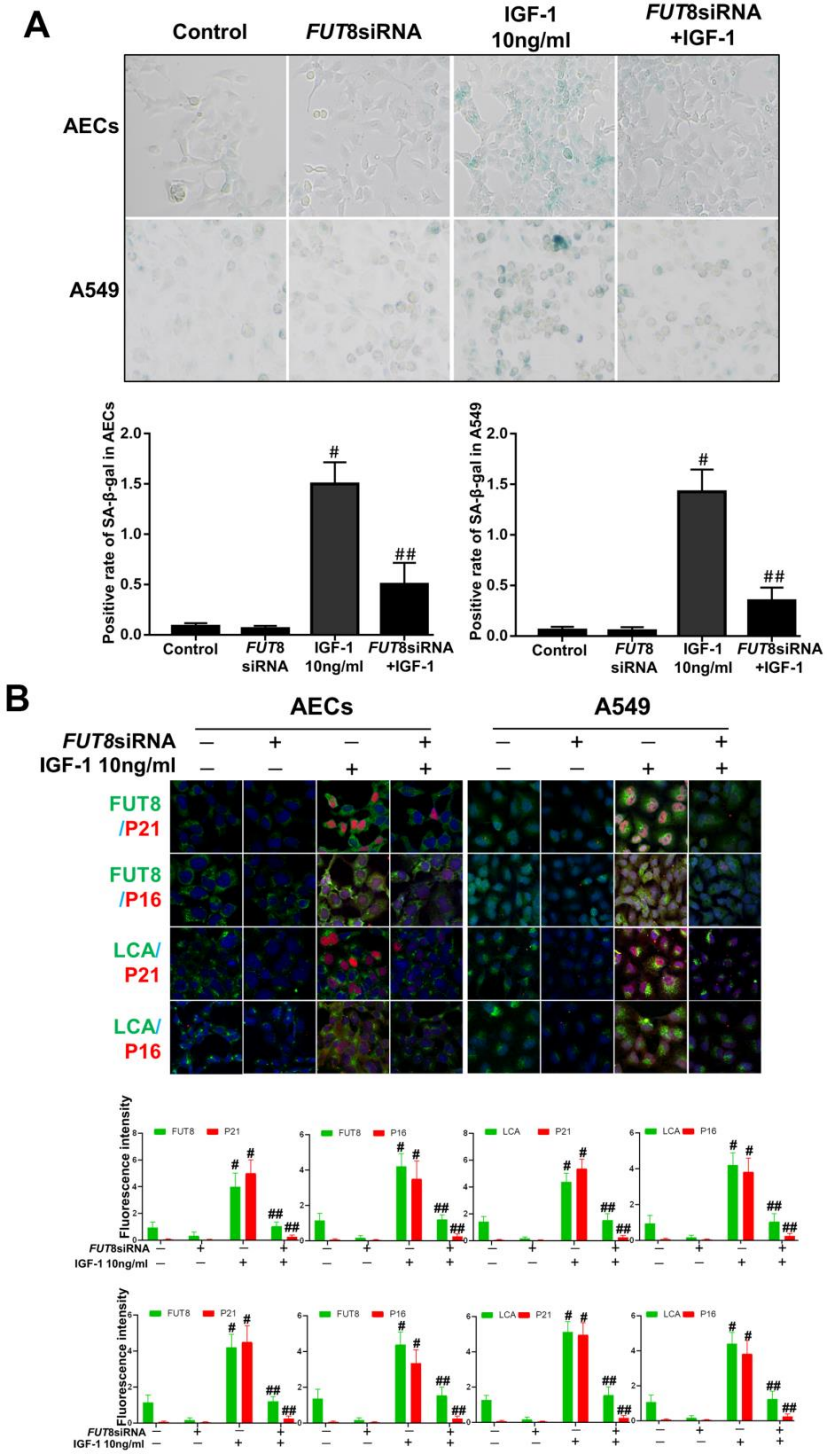
**AEC senescence was inhibited upon *FUT8* knockdown in *vitro*.** (**A**) SA-β-gal staining was performed to detect cellular senescence of different groups (original magnification, 200×). (**B**) Representative images of dual staining for FUT8 (green) and P21 (red) staining, FUT8 (green) and P16 (red) staining, LCA (green) and P21 (red) staining and LCA (green) and P16 (red) staining are shown *in vitro* (original magnification, 200×). Data are shown as the mean ± SEM, n ≥ 3 per group. #*P* < 0.01, ##*P* < 0.01. #Indicates the comparison of the control group with the IGF1 group; ##indicates the comparison of the *FUT8* siRNA+ IGF1 group with the IGF1 group. One-way ANOVA followed by Dunnett’s Multiple Comparison Test.

**Figure 5:**
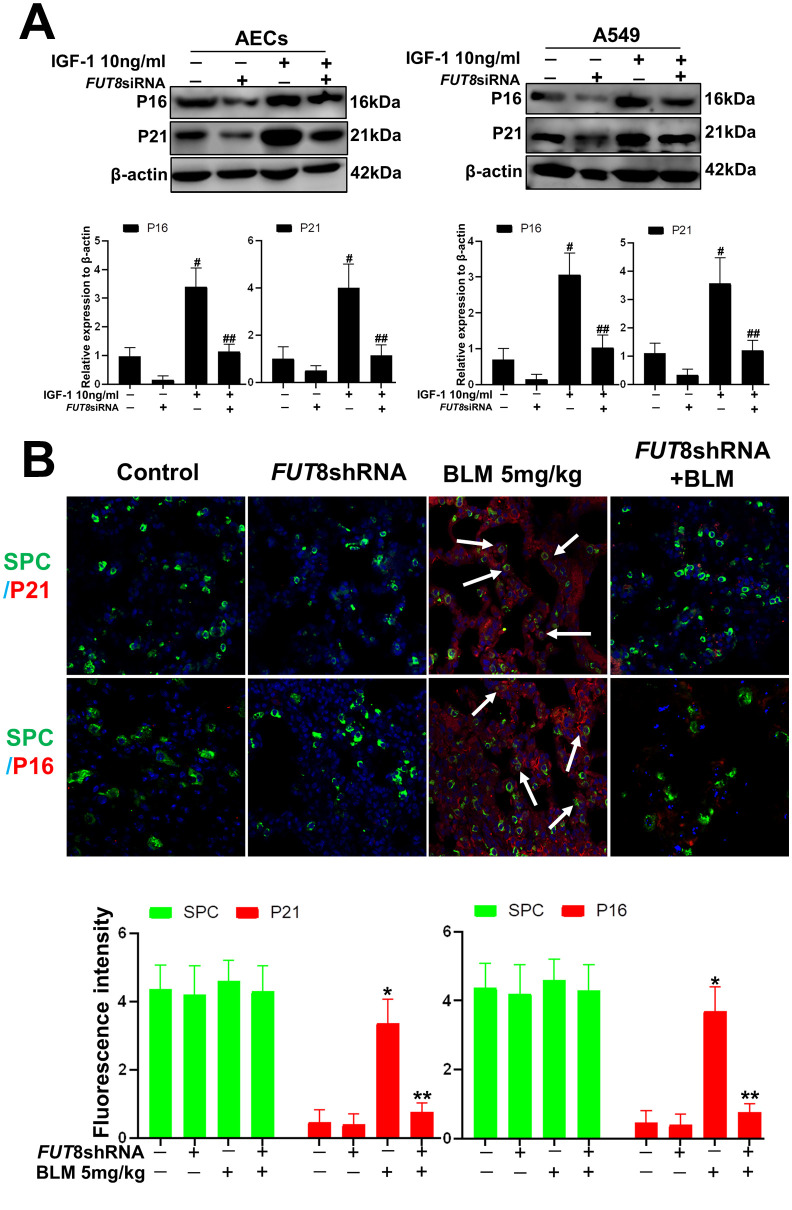
**AEC senescence was inhibited upon *FUT8* knockdown *in vitro* and *vivo*.** (**A**) P21 and P16 levels were assessed using western blotting analyses in different groups. (**B**) Representative immunofluorescence images of dual staining for SPC (green) and P21 (red), SPC (green) and P16 (red) are shown *in vivo* (original magnification, 200×). Data are shown as the mean ± SEM, n ≥ 3 per group. #*P* < 0.01, ##*P* < 0.01, *P < 0.01, **P < 0.01. #Indicates the comparison of the control group with the IGF1 group; ##indicates the comparison of the *FUT8* siRNA+ IGF1 group with the IGF1 group. *indicates the comparison of the control group with the BLM group; **indicates the comparison of the *FUT8*shRNA+ BLM group with the BLM group. One-way ANOVA followed by Dunnett’s Multiple Comparison Test.

*In vitro*, the AECs senescence model was subjected to a small interfering RNA (siRNA) to silence *FUT8* expression (Supplementary Figure 1). The effects of downregulation of CF on the AECs senescence induced by IGF1 were then observed. We found that the rate of positive SA-β-Gal staining decreased after *FUT8* knockdown (Figure 4A). The results of immunofluorescence showed that the decreased p21, p16 accompanied by downregulated FUT8 and LCA levels after *FUT8* knockdown (Figure 4B). Western blotting showed that AECs senescence induced by IGF1 was substantially alleviated after *FUT8* knockdown, along with decreased levels of P21, P16, FUT8 (Figure 5A). This suggested that down regulating CF can block AECs senescence induced by IGF1.

To further confirm the role of CF *in vivo*, we knocked down *FUT8* to evaluate subsequent AECs senescence. The *FUT8*shRNA was packaged using adenovirus and injected into the tail vein of mice to silence *FUT8* expression as described in our previous research. The knocked down effects were verified using RT-PCR and western blotting (Supplementary Figure 2). The results of immunofluorescence showed that silencing of *FUT8* dramatically decreased the levels of P21 and P16 in SPC-labeled AECs (Figure 5B).

### Downregulation of CF decreases the levels of CTGF, TGFβ1, and MMP9 in SASP induced by IGF1

**Figure 6:**
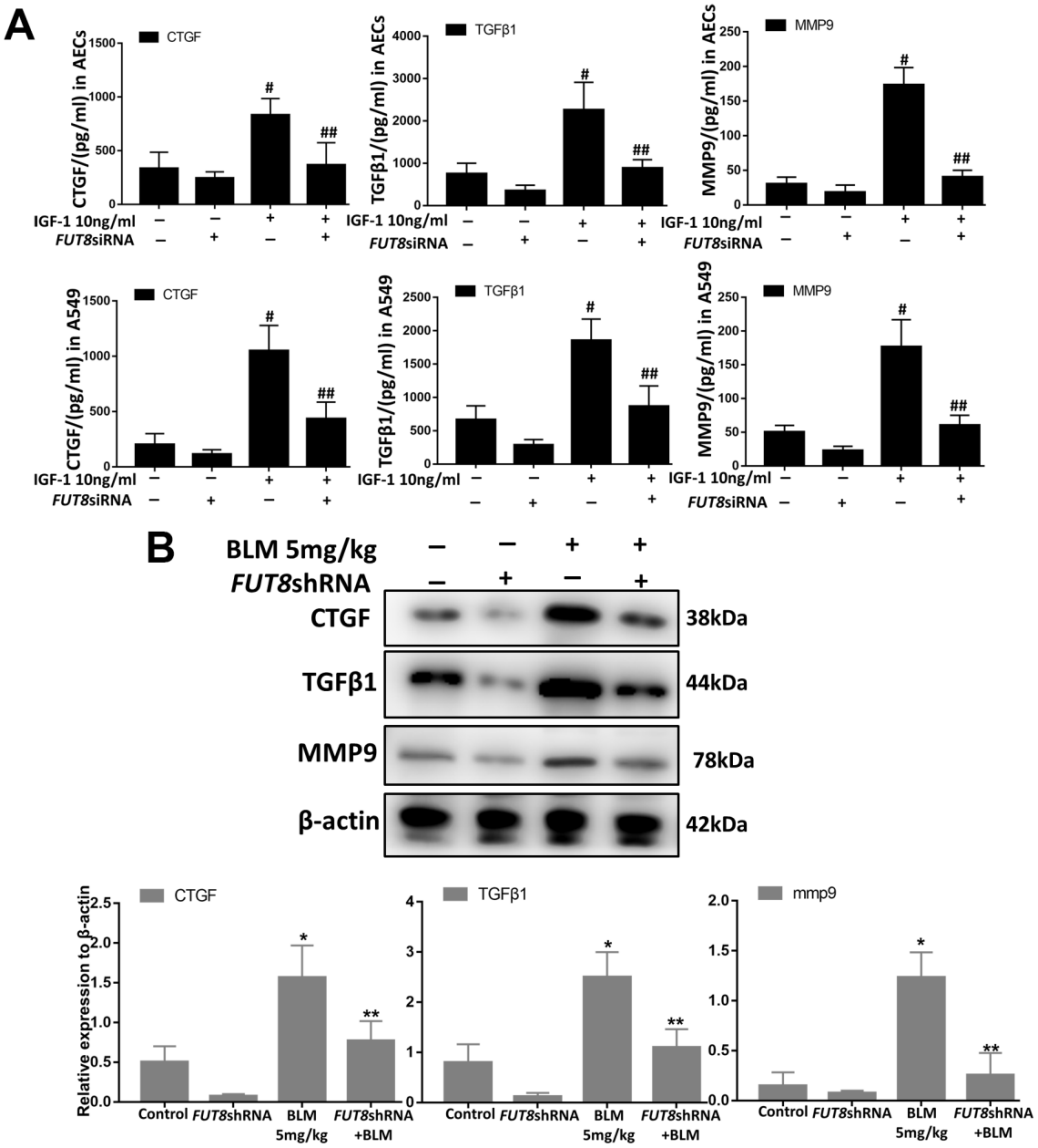
**CTGF, TGF1, and MMP9 were inhibited upon FUT8 knockdown *in vitro* and *in vivo*.** (**A**) A549 and AECs were stimulated by IGF1 for 72 h, the supernatants were collected, and the levels of CTGF, TGF 1, and MMP9 were measured using ELISA. #P < 0.01, ##P < 0.01. #Indicates the comparison of the control group with the IGF1 group; ## indicates the comparison of the FUT8siRNA+ IGF1 group with the IGF1 group. (**B**) CTGF, TGF1, and MMP9 levels were assessed using western blotting analyses *in vivo*. *P < 0.01. **P < 0.01. * indicates the comparison of the control group with BLM group; ** indicates the comparison of the FUT8shRNA+ BLM group with the BLM group. Data are shown as the mean ± SEM, n ≥ 3 per group. One-way ANOVA followed by Dunnett’s Multiple Comparison Test.

Senescence is often accompanied by senescence associated secretory phenotype (SASP). The results of an enzyme-linked immunosorbent assay (ELISA) showed that the levels of CTGF, TGFβ1, and MMP9 were higher in IGF1-stimulated AECs and in A549 culture supernatants, and CF inhibition by silencing *FUT8* reduced the secretion of the above cytokines (Figure 6A). Western blotting showed that the levels of CTGF, TGFβ1, and MMP9 in mouse lung tissue were upregulated significantly by BLM, and their levels were downregulated after inhibition of CF by *FUT8* silencing (Figure 6B). These results suggested that inhibition of CF modification can reduce the secretion of CTGF, TGFβ1, and MMP9 in SASP induced by IGF1.

### Downregulation of CF alleviates lung fibrosis induced AEC senescence by IGF1

**Figure 7:**
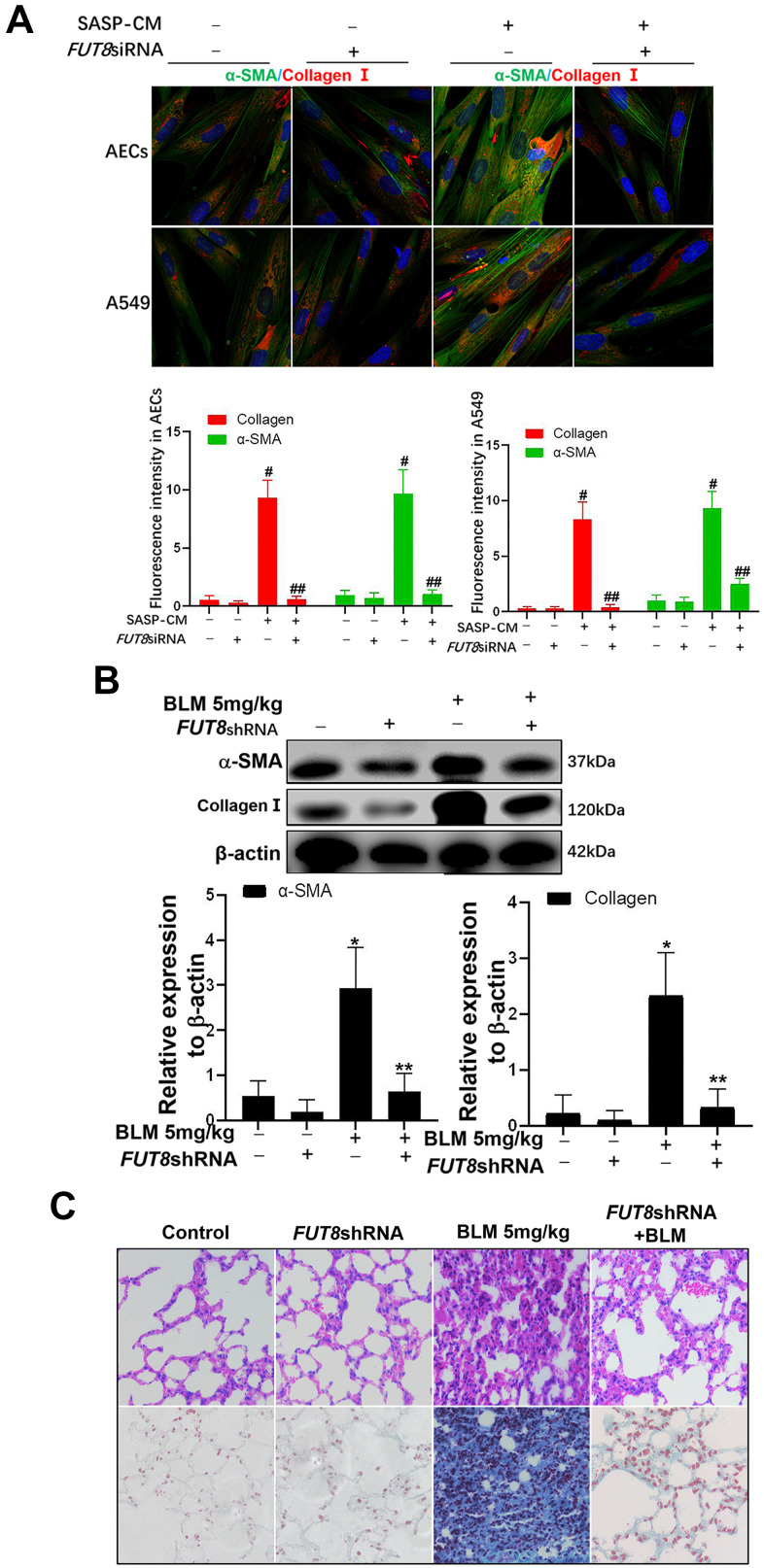
**The fibroblast transition into myofibroblasts induced by the supernatants of senescent AECs can be inhibited by *FUT8* knockdown.** (**A**) After IGF1 was added to induce AEC senescence for 72 h, the medium was replaced by fresh medium without IGF1 to culture for another 3 days, then the supernatants were collected to culture MRC5 cells. The expression of α-SMA (green) and collagen I (red) were investigated using immunofluorescence (original magnification, 200×). (**B**) The expression of fibrotic markers, α-SMA and collagen I, were investigated by western blotting *in vivo*. (**C**) Representative images of HE and Masson’s trichrome-stained BLM lung sections (original magnification, 200×). #*P* < 0.01, ##*P* < 0.01. #Indicates the comparison of the control group with the SASP-CM group; ##indicates the comparison of the *FUT8*siRNA+ SASP-CM group with the SASP-CM group. *P < 0.01, **P < 0.01. * indicates the comparison of the control group with the BLM group; ** indicates the comparison of the *FUT8*shRNA+BLM group with the BLM group. Data are shown as the mean ± SEM, n ≥ 3 per group. One-way ANOVA followed by Dunnett’s Multiple Comparison Test.

In SASP induced by IGF1, CTGF, TGF-β1, and MMP9 are important cytokines for the transformation from fibroblasts to myofibroblasts in the lung interstitium. The supernatants of senescent A549 and AECs were collected to culture the fibroblast cell line MRC-5 for 3 days. We found that the levels of α-SMA and collagen I, which are hallmarks of myofibroblasts, were both higher in MRC-5 cells cultured in senescent conditioned medium (SASP-CM) compared with those in cells cultured control conditioned medium (CCM). The levels of α-SMA and collagen I were downregulated significantly after the CF was decreased via *FUT8* silencing in the SASP-CM group (Figure 7A). Western blotting showed that downregulation of CF reduced the levels of α-SMA and collagen I in the BLM mouse model and alleviated pulmonary fibrosis (Figure 7B, 7C).

### CF is important regulatory target of IGF1/PI3K/AKT signaling pathway

**Figure 8:**
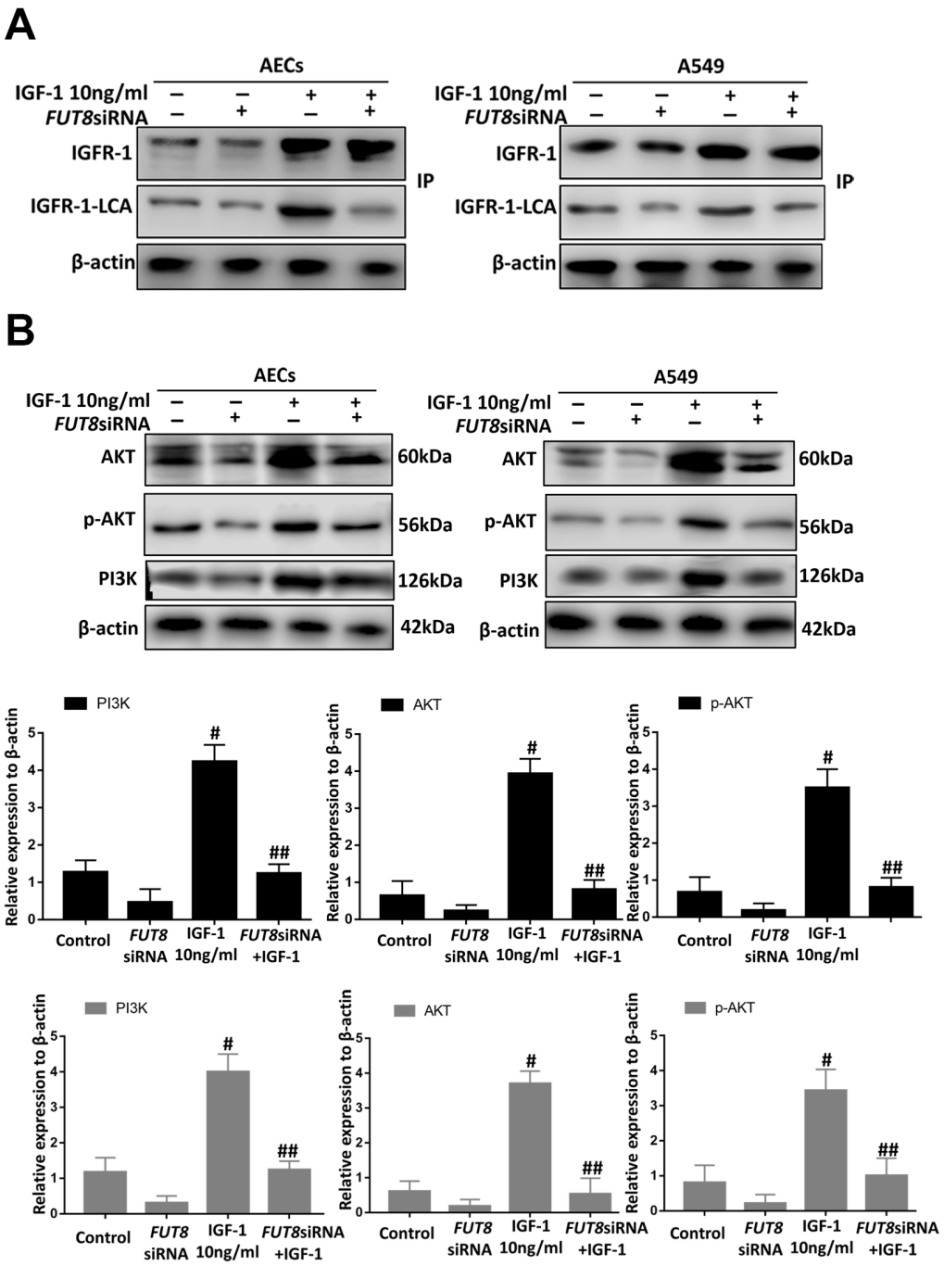
**CF regulates AEC senescence through the IGF1/PI3K/AKT pathways *in vitro*.** (**A**) The IGFR-1 level in total cell lysates was assessed using western blotting analyses. Lectin blot analysis of the immunoprecipitated IGFR-1 protein. IGFR-1 was immunoprecipitated from whole cell lysates using anti-IGFR-1 antibodies. The blots were probed with LCA. Representative data are shown. Quantification is shown in the lower panel. (**B**) PI3K, AKT and p-AKT levels were assessed using western blotting analyses. Total cell lysates were subjected to immunoblotting. ^#^*P* < 0.01, ^##^*P* < 0.01, ^#^ indicates the comparison of the control group with the IGF1 group; ^##^ indicates the comparison of the *FUT8*siRNA+ IGF1 group with the IGF1 group. One-way ANOVA followed by Dunnett’s Multiple Comparison Test. Each experiment was performed in triplicate.

Immunoprecipitation of LCA showed that CF of IGFR-1 increased significantly after IGF1 induction over time and was alleviated by *FUT8* knockdown *in vitro* (Figure 8A). Moreover, the levels of phosphorylated PI3K, AKT and p-AKT decreased significantly in response to *FUT8* knockdown (Figure 8B). These results suggested that in AECs senescence, activation of IGF1/PI3K/AKT signaling depended on CF.

## DISCUSSION

According to the clinical epidemiological data (i.e., IPF mainly affects elderly people), most of the literature reports and our studies support the view that IPF is an aging-related lung disease and is associated with the AECs senescence [23]. AECs have robust regeneration and repair ability under pathological conditions, such as chronic injury; however, the repair ability of AECs is mostly lost when IPF occurs [24]. Recent studies have found that senescence is an important reason for the decline of the repair function of AECs [25]; however, the regulatory mechanism of AECs senescence is not clear. The present study made two discoveries: 1) The IGF1 signaling pathway participates in the pathogenesis of IPF by inducing AECs senescence; and 2) CF acts as a target to regulate IGF1 signaling pathway-mediated activation of AECs senescence.

Activation of the IGF1/PI3K/AKT signaling pathway is an important mechanism in senescence [11]. Indeed, by evaluating the levels of key proteins in IPF lung tissue, we found a significant upregulation of IGF1, PI3K, AKT and p-AKT, indicating activation of this signaling pathway in IPF. However, whether this signaling pathway is involved in AECs senescence and how this signaling pathway is regulated in IPF are unclear. Our previous study observed an increased level of CF in a BLM-induced mouse model of IPF [22]. IGFR-1, a member of the IGF1 signaling pathway, is a glycoprotein with post-translational sugar modification [20]. Therefore, we speculated that CF might be an important target for the precise regulation of the IGF1 signaling pathway. In this study, we observed that AECs in IPF lung tissue showed extensive aging and upregulated CF.

The cellular senescence theory states that aging cells accumulate damage and affect their own repair function [26, 27]. However, senescence is not simply the result of the increasing age, but also the result of the innate reaction under pathological conditions, such as chronic injury [28]. In recent decades, IGF1 has become a research focus, not only because inactivating the IGF1 signaling pathway can prolong life, but also because activating this signaling pathway would release inflammatory factors, aggravate the oxidative stress response and other pathological conditions, and jointly accelerate cell senescence [29]. However, it is not clear whether IGF1 is an important cause of AEC senescence in IPF. Therefore, IGF1 was administrated to AECs *in vitro*, which promoted AEC senescence, as expected. Interestingly, we found that CF levels were elevated significantly during AECs senescence induced by IGF1 stimulation. Subsequent downregulation of CF reduced the number of senescent AECs significantly. These findings suggest that CF affects IGF1-induced AECs senescence.

Unlike other SASP profiles, CTGF, TGFβ1 and MMP9 are important cytokines for IPF development [14–16]. CTGF promotes the activation and proliferation of fibroblasts, collagen synthesis, and the secretion of TGFβ1, which causes fibroblasts to transform into myofibroblasts [30]. MMPs can activate the inflammatory signaling pathway and promote the accumulation of inflammatory cells and ECM in the lung interstitium [31]. In this study, the levels of CTGF, TGFβ1, and MMP9 increased significantly after AECs senescence was induced by IGF1, and myofibroblasts accumulated in the lung tissue. Down-regulation of CF significantly decreased the levels of these cytokines, blocked the aggregation of myofibroblasts, and alleviated pulmonary fibrosis in mice. These findings demonstrated that the downregulation of CF could inhibit the profibrotic effects of IGF1-induced AECs senescence.

CF has been shown to affect the binding between receptors and ligands, and further regulates the activity of downstream signaling pathways [19]. Therefore, we hypothesized that CF might be a regulatory target to prevent AECs senescence induced by IGF1/PI3K/AKT signaling pathway activation. We found that activation of the IGF1/PI3K/AKT signaling pathway was dependent on LCA on the IGFR-1 surface during IGF1-induced AECs senescence. Downregulation of CF could block the expression of downstream phosphorylated proteins by inhibiting LCA on the IGFR-1 surface.

AECs senescence is caused by various factors [32]. The balance of the lung interstitial micro-environment is precisely regulated by elaborate adjacent structures between alveoli and blood vessels, and by cross-talk among cells [33]. Once the micro-environment balance is disrupted, AEC senescence is initiated [34]. Cross-talk, such as that among IGF1, TGF-β, and Wnt, can also induce AECs senescence [35–37]. Blockade of a single signaling pathway can inhibit IPF to some extent; however, other pathways would be activated in compensation. Whether there is activation of other signaling pathways in AECs senescence and whether CF can be a target in the activation of other signaling pathways requires further study.

In conclusion, this study identified a potential treatment target for IPF progression caused by AECs senescence, which will help to understand and prevent aging-related diseases, and allow the development of anti-IPF drugs.

## MATERIALS AND METHODS

### Collection of samples from patients with IPF

The samples of lung tissues from patients with IPF (n = 6) were obtained from the Department of Lung Transplantation, The First Affiliated Hospital of Guangzhou Medical University, Guangzhou, China (Table 1). All diagnoses of IPF were made in accordance with the European Respiratory Society (ERS) and American Thoracic Society (ATS) criteria for IPF 2015. Normal peripheral tissues (n = 6) used as controls were obtained from patients with tumors from the Thoracic Surgery Department of Peking Union Medical College Hospital, Chinese Academy of Medical Sciences. Informed consent was obtained from patients, and the study was approved by the Ethics Committee of Peking Union Medical College Hospital (JS-1127).

**Table 1:** Characteristics of the patients with idiopathic pulmonary fibrosis categorized according to the 2015 IPF statement.

**Patients characteristics**	**IPF (N=6)**	**None-IPF(N=6)**	***P*-value**
Gender (F/M)	1/5	2/4	0.999
Age (year)	73.7±6.5	65.3±13.7	0.205
BMI (kg/m^2^)	26.9 ±4.3	27.7±6.0	0.796
Pack-years	41.2±14.3	36.2±10.6	0.506

Numbers are presented as mean ±SD. Abbreviations: IPF, idiopathic pulmonary fibrosis; BMI, body mass index.

### Cell lines and the isolation and culture of primary AECs in mice

A549 and MRC5 cells were purchased from the American Type Culture Collection (ATCC, Manassas, Virginia, USA). The cells were cultured in Dulbecco’s modified Eagle’s medium (DMEM) containing 1% penicillin/streptomycin and 10% fetal bovine serum (FBS) at 37° C under 5% CO_2_.

Six-to eight-week-old male SPF C57BL/6 mice (Laboratory Animal Center, Peking Union Medical College Hospital, Beijing, China) were sacrificed humanely, and their lungs were removed, washed using phosphate-buffered saline (PBS) three times, and cut into very small pieces (like fine sand). Collagenase I (Gibco, Life Technologies) 200 U/mL was added to digest the lung tissues for 30 min at 37° C, and the reaction was terminated with DMEM containing 10% FBS. After washing three times using PBS, the cell suspension was transferred into culture flasks coated with mouse IgG-supplemented complete medium. After 2 hours, the detached cells were removed by centrifugation, and the supernatants were discarded. Pellets were suspended in complete medium and cultured in a 37° C humidified incubator containing 5% CO_2_. Finally, immunofluorescence staining for SPC, E-cadherin (Abcam, Cambridge, MA, USA) was conducted to identify AECs.

### Animal care and mouse pulmonary fibrosis models

Male C57BL/6 mice (six-to eight-weeks-old, specific pathogen free) were obtained from the Laboratory Animal Center, Peking Union Medical College Hospital and housed at a constant room temperature with a 12 h light/dark cycle. Standard rodent chow and water were provided *ad libitum*. Animal experiments were conducted in accordance with the regulations established by the Institutional Committee for the Care and Use of Laboratory Animals and were approved by the Chinese Academy of Medical Sciences Laboratory Animal Center; all efforts were made to minimize suffering. All surgical procedures were conducted by a single surgeon under aseptic conditions in the Laboratory Animal Unit. The mice were injected intratracheally with 50 μl of 5 mg/kg bleomycin. On day 14 after bleomycin treatment, the mice were sacrificed, and lungs were collected for subsequent experiments.

### Immunofluorescence analysis of IPF lung tissues

Lung tissues were fixed using 4% paraformaldehyde, paraffin-embedded, and cut into 2-μm sections. The sections were placed on polylysine-coated slides and incubated in a 60° C oven. After dewaxing with xylene, the sections were rehydrated using an alcohol concentration gradient. The slides were then placed in a microwave oven in antigen retrieval solution and incubated at 100° C for 10 min once, for 2 min four times, cooled to room temperature for 20 min, and then washed with PBS for 5 min. Primary antibodies against P21 (Abcam) and FUT8 (Abcam) were incubated with the slides overnight at 4° C. Secondary antibodies and LCA (Vector Labs, Burlingame, CA, USA) were incubated with the samples for 1 h at room temperature. Then, sections were mounted with Fluorescent Mounting Media containing 4′,6-diamidino-2-phenylindole (DAPI). Each tissue section was observed under a confocal laser scanning microscope at magnifications of ×200. Negative controls did not receive the first antibody.

### Immunofluorescence staining *in vitro*

AECs and A549 cells were fixed with freshly prepared 4% paraformaldehyde for 10 min at room temperature. The cells were then washed three times with PBS and mounted on coverslips. Each coverslip was then incubated in 1% BSA. Primary antibodies against P21 (Abcam) and FUT8 (Abcam) were incubated with the cells overnight at 4° C. After washing with PBS, LCA (Vector Labs) and secondary antibodies were incubated with the cells for 1 h at room temperature in a darkened humidified chamber. Finally, each cover slip was washed with PBS, and mounted in fluorescent mounting medium with DAPI. Images were acquired using a confocal laser scanning microscope at a magnification of ×200.

### Immunofluorescence staining of mouse lung tissues

Mouse lung tissues were fixed with 4% paraformaldehyde and then cut into 4-μm sections. After dehydration, 4-μm cryosections were collected on Superfrost Plus glass slides. Sections were rinsed with PBS and permeabilized with a 1% Triton solution for 5 min. They were then blocked with 1% BSA for 1 h. Primary antibodies against FUT8 (Abcam), P21 (Abcam), and SPC (Abcam) were incubated with sections overnight at 4° C. LCA (Vector Labs) and secondary antibodies were incubated with the samples for 1 h at room temperature. The sections were mounted with Fluorescent Mounting Media with DAPI. Each tissue section was observed under a confocal laser scanning microscope at a magnification of ×200.

### Real-time PCR

Total RNA was extracted from the cells and lung tissues using TRIzol according to the manufacturer’s instructions. The total RNA was subjected to an RT reaction with the SYBR PrimerScript RT-PCR Kit according to the manufacturer’s instructions. Relative levels of mRNA for FUT8 were determined by real-time PCR with a LightCycler (Roche, Mannheim, Germany) according to the manufacturer’s manual. The targeted genes were amplified with the following primers: 5’-TGGACTGCACAATCGATACACGA-3’(forward) and 5’-AGTTTGCAGAGGCATCAGGATGTAG-3’(reverse), were for FUT8 (A549); 5′ CTGGT-TCCTGGCGTTGGATT-3’(forward) and 5′CTCAGCCATTCGCCTCAAGT 3’ (reverse), were for FUT8(AECs and mice); 5’-GAAGGTGAAGGTCGGAGT-3’ (forward) and 5’-GAAGATGGTGATGGGATTTC3’ (reverse) were for GAPDH.

### Senescence-associated β-galactosidase staining

The Senescence β-Galactosidase Staining Kit was purchased from Cell Signaling Technology (Danvers, MA, USA). Cell samples on 6-well dishes were fixed with 4% formaldehyde for 10 min at room temperature. The cells were washed three times with PBS for 5 min and then incubated with freshly prepared SA-β-Gal staining solution overnight in a 37° C. The next day, the stained cells imaged under a Zeiss Axio Observer inverted microscope using differential interference contrast brightfield microscopy (DIC). For each slide, at least three fields were captured to calculate the SA-β-gal intensity.

### Inhibition of *FUT8* expression

We constructed an adenovirus carrying the *FUT8*shRNA in our previous study. The adenovirus carrying the *FUT8*shRNA was administered by tail vein injection of 1 × 106 plaque forming units (PFUs) before BLM modeling. The *FUT8*siRNA was designed by GeneCopoeia (Rockville, MD, USA). The sequences of the siRNA targeting human *FUT8* were as follows: 5′-GCCGAGAACTGTCCAAGATTC-3′. The sequences of the siRNA targeting mouse *FUT8* were as follows: 5′-GCUACUGAUGAUCCUACUUdTdT-3′. a siTran 1.0 transfection reagent (OriGene Technologies) was used for transfection, according to the manufacturer’s protocol. Twenty-four hours after the transfection, A549 and AECs were incubated with serum-free medium for 24 h. To confirm the knockdown of *FUT8*, we extracted protein from cells and assessed FUT8 levels using western blotting.

### Western blotting analysis

Total proteins were collected according to the methods described in the manufacturer's instructions (Keygene, Shanghai, China). The protein concentration was assessed using a Thermo Fisher BCA kit (Thermo Fisher Scientific, Waltham, MA, USA). Protein samples were separated by 10% SDS-PAGE and transferred to a polyvinylidene fluoride (PVDF) membrane. The samples were incubated with primary antibodies overnight at 4° C. The membranes were incubated with a horseradish peroxidase (HRP)-conjugated secondary antibody at room temperature for 1.5 h, and the immunoreactive protein bands were detected using a chemiluminescence device.

### Lectin blotting

Immunoprecipitated IGFR-1 was separated on 12% SDS-PAGE gels and electrotransferred to PVDF membranes. The membranes were blocked with 5% BSA in Tris-buffered saline containing 0.05% Tween 20 (TBST) overnight at 4° C, and then incubated with TBST containing LCA-Biotin (Vector Labs), which preferentially recognizes Fuc-1,6GlcNAc, for 1 h at 23° C. Blots were washed three times with a 1× PBS-Tween solution and incubated with ECL reagents (Amersham, Pittsburgh, PA, USA) for 1 min. The lectin-reactive proteins were visualized using super RX-N film (Fujifilm Corporation, Tokyo, Japan).

### ELISA

The levels of MMP9, TGFβ1, CTGF in supernatants were measured using commercially available ELISA kits (Raybiotech, Peachtree Corners, GA, USA), according to the manufacturers’ instructions.

### Statistical analysis

The results are presented as means ± standard deviations. Values were compared using an unpaired two-tailed Student’s t-test to compare two groups. Differences among more than two groups were assessed using one-way analysis of variance (ANOVA). A value of *P* < 0.05 was considered to indicate statistical significance. All statistical analyses were performed using the statistical package SPSS (version 21.0, IBM Corp., Armonk, NY, USA).

## Supplementary Material

Supplementary Figures
