# Supplementary material for: Regulation of the IGF1 signaling pathway is involved in idiopathic pulmonary fibrosis induced by alveolar epithelial cell senescence and core fucosylation
Source: Aging (Albany NY). 2021 Jul 30;13(14):18852–69. doi: 10.18632/aging.203335 (PMC8351684; doi:10.18632/aging.203335)
Supplement: Supplementary Figures [file aging-13-203335-s001.pdf]

SUPPLEMENTARY FIGURES

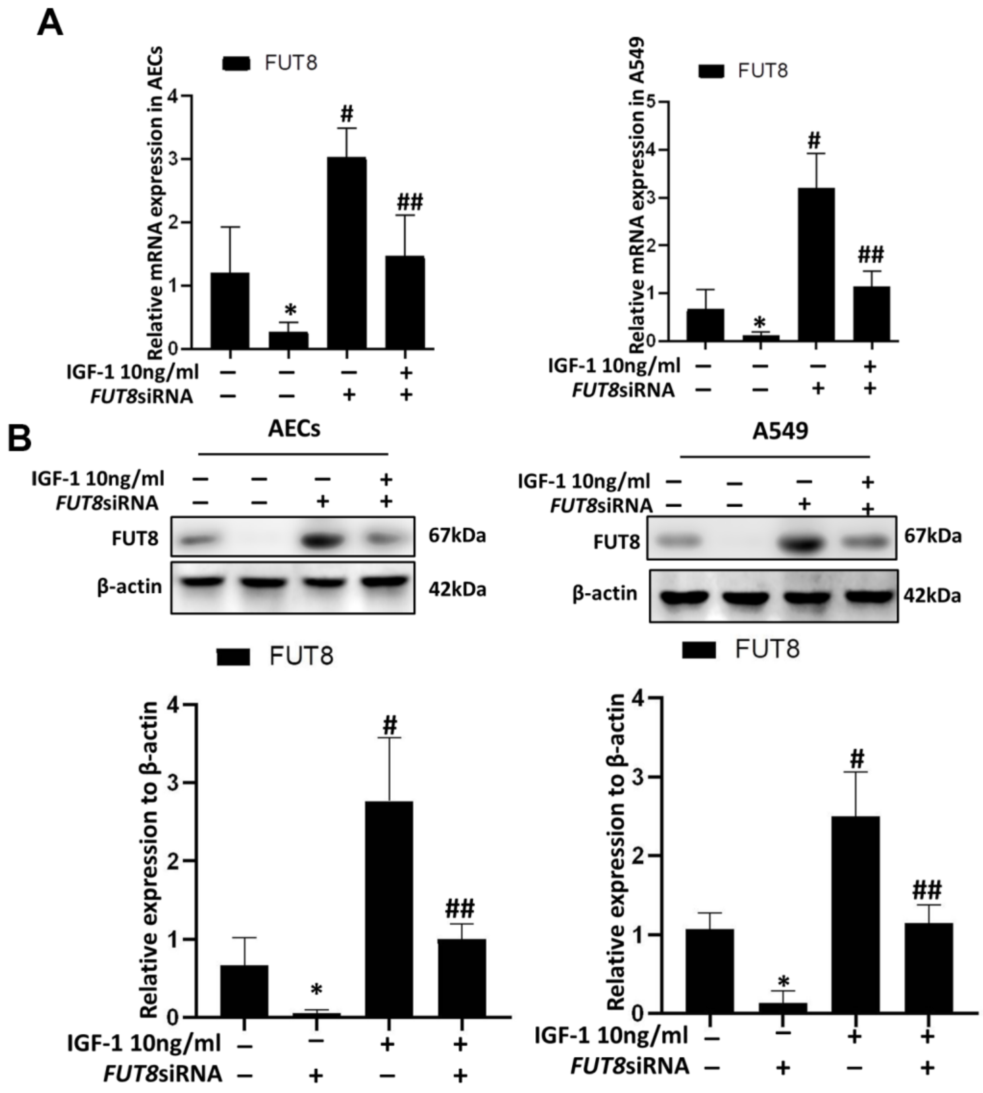

Supplementary Figure 1. Effect of FUT8siRNA transfection on core fucosylation in AECS and A549 cells. (**A**) RT-PCR indicate that FUT8siRNA inhibited the mRNA of endogenous FUT8. (**B**) Western blotting indicate that FUT8siRNA inhibited the protein expression of endogenous FUT8. Data are shown as the mean ± SEM, n ≥ 3 per group. *P < 0.01, #P < 0.01, ##P < 0.01. *Indicates the comparison of the FUT8siRNA group with control group; # indicates the comparison of the control group with the IGF1 group; ##indicates the comparison of the FUT8 siRNA+ IGF1 group with the IGF1 group. One-way ANOVA followed by Dunnett's Multiple Comparison Test.

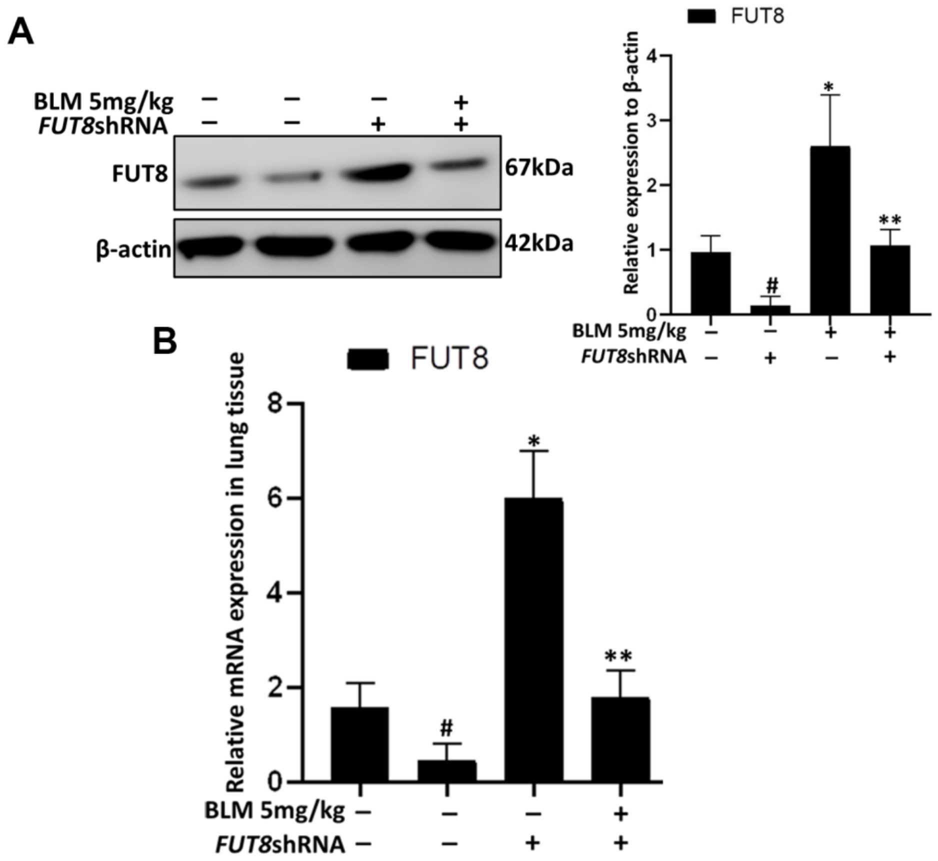

Supplementary Figure 2. *FUT8*shRNA effectively inhibits endogenous a-1,6 fucosyltransferase (FUT8) expression in the lung tissues. (**A**) Representative RT–PCR analysis of FUT8 mRNA expression in the BLM induced mouse model lung infected with FUT8shRNA. (**B**) Representative western blot analysis and quantification of FUT8 protein expression in the BLM induced mouse model lung infected with *FUT8*shRNA. Data are shown as the mean ± SEM, n ≥ 3 per group. #*P* < 0.01, **P* < 0.01, ***P* < 0.01. # Indicates the comparison of the *FUT8*shRNA with control group; * indicates the comparison of the control group with the BLM group; ## indicates the comparison of the *FUT8*shRNA+BLM group with the BLM group. One-way ANOVA followed by Dunnett's Multiple Comparison Test.
